# Obesity-driven metabolic reprogramming and immune dysfunction in renal cancer

**DOI:** 10.3389/fimmu.2026.1866875

**Published:** 2026-06-11

**Authors:** Henry N. Ogbonna, Lyse A. Norian

**Affiliations:** 1 Graduate Biomedical Sciences, Pathology, Pharmacology, and Physiology Theme, University of Alabama at Birmingham, Birmingham, AL, United States; 2 Department of Microbiology, Immunology, and Molecular Genetics, University of Kentucky, Lexington, KY, United States; 3 Markey Cancer Center, University of Kentucky, Lexington, KY, United States

**Keywords:** adipokines, clear cell renal cell carcinoma, immune checkpoint inhibitors, immunometabolism, lipid metabolism, myeloid cells, obesity, obesity paradox

## Abstract

Renal cell carcinoma (RCC), specifically clear cell renal cell carcinoma (ccRCC), is a metabolic tumor wherein the physiological state of the host is central to tumor development, progression, and therapeutic resistance. Obesity has emerged as one of the major risk factors associated with RCC; however, its impact on RCC is more complex than simply the accumulation of excess body fat. Obesity transforms the renal tumor microenvironment through metabolic rewiring and alterations in inflammation, vasculature, and anti-tumor immunity. The expansion of adipose tissue in obesity alters the renal microenvironment through the production of fatty acids, adipokines, and cytokines in a manner that not only supports tumor growth but also promotes immunosuppression. Increased levels of leptin, resistin, IL-1β, IL-6, IL-8, and VEGF - together with decreased levels of adiponectin and omentin-1 - promote angiogenesis, stromal remodeling, recruitment of myeloid cells, and evasion of immune checkpoint inhibition. These obesity-driven factors interact with the intrinsic metabolism of ccRCC cells, including lipid accumulation, glycolysis, hypoxic signaling, and metabolic plasticity. Furthermore, obesity reshapes the immune environment through recruitment of MDSCs, polarization of TAMs, dysfunction of DCs, neutrophil-mediated immunosuppression, T cell exhaustion, and increased abundance of regulatory T cells, reinforcing an immunosuppressive state. These effects of obesity in RCC are particularly relevant in the context of the obesity paradox, wherein obesity has been associated with improved treatment outcomes, which are not uniformly observed across RCC cohorts. These differences may reflect the limitations of body mass index as a biological indicator of obesity, together with variations in systemic inflammation, body composition, and treatment context. Here, we summarize current knowledge on obesity-driven immunometabolic rewiring in RCC and outline key priorities for the field, including obesity-relevant preclinical models, biomarkers of visceral adiposity and systemic inflammation, and clinical trials targeting immunometabolism.

## 1 Introduction

Renal cell carcinoma, a malignant tumor of epithelial cell origin in the nephron, is the most common form of adult primary renal cancer, accounting for 80-85% of total renal cancer cases (1, 2). Statistics on cancer incidence and mortality worldwide indicate that approximately 434, 840 new cases of kidney cancer occur annually, resulting in 155, 953 deaths; thus, kidney cancer accounts for 2.2% of total cancer incidence and 1.6% of total cancer-associated mortality worldwide (1, 2). RCC includes various subtypes of renal epithelial cell cancer, such as clear cell carcinoma, papillary carcinoma, and chromophobe carcinoma (3). Of these, clear cell RCC - characterized by an accumulation of intracellular lipid and glycogen droplets - is the predominant subtype, comprising over 70% of RCC cases, followed by papillary RCC (15%), and chromophobe RCC (5%). Additional uncommon histologic variants represent the remainder of RCC cases (4, 5).

At a population level, RCC remains one of the most lethal genitourinary malignancies. Despite advances in imaging and early detection, many patients are still diagnosed with locally advanced or metastatic RCC, making treatment more difficult (6). Due to its prevalence, clear cell RCC forms the basis for current systemic treatment strategies (7). The first-line treatment for localized clear cell RCC is surgery, followed by adjuvant immune checkpoint inhibitors (ICI) like pembrolizumab (anti-PD-1) to reduce recurrence in high-risk cases. For patients with advanced or metastatic clear cell RCC, the first-line treatment includes immuno-oncology (IO)-based combinatorial therapies (8) that typically include ICI, such as pembrolizumab (anti-PD-1), used alongside tyrosine kinase inhibitors (TKIs) that block vascular endothelial growth factor receptor (VEGFR) signaling, such as axitinib, lenvatinib, and cabozantinib. In the later stages of clear cell RCC, second-line treatment options include VEGFR TKIs, mechanistic target of rapamycin (mTOR) inhibitors, and belzutifan, a hypoxia inducible factor-2α (HIF-2α) inhibitor (8). Despite the numerous treatment options now available, recurrence after curative-intent treatment remains common, with about 30% of patients relapsing after apparently localized disease, and long-term survival for metastatic disease remains poor, with a 5-year relative survival of 20.3% for distant-stage kidney cancer (9–11).

A mounting body of evidence indicates that host metabolism is a key modifier of RCC development, biology, and IO-based treatment efficacy. An individual’s metabolic status shapes nutrient availability, systemic and local inflammation, and immune tone in parallel with oncogenic signaling (12, 13). Clear cell RCC, in particular, is characterized by alterations in lipid metabolism, and angiogenesis- and hypoxia-related signaling cascades, all of which are modified by a patient’s metabolic status. Clear cell RCC is microscopically identified by the presence of intracellular lipid and glycogen deposits. These cellular alterations can be seen as the consequence of the overall reprogramming of lipid uptake and storage mechanisms (14). Lipidomic analyses have shown that clear cell tumors exhibit elevated levels of cholesterol, cholesterol esters, and triglycerides compared to non-malignant renal tissue (15). Moreover, clear cell RCC suppresses intrinsic cholesterol biosynthesis while increasing dependence on cholesterol import, particularly through receptors such as scavenger receptor class B type 1 (SCARB1) (16). Inactivation of the von Hippel–Lindau (VHL) gene is a defining event in clear cell RCC, as it disrupts the normal degradation of hypoxia-inducible factors (HIFs), resulting in continuous activation of hypoxia-responsive genes even in oxygen-rich environments (17). This dysregulated VHL-HIF pathway translates metabolic changes into enhanced tumor angiogenesis by upregulating pro-angiogenic growth factors involved in tumor vessel formation, including VEGF and platelet-derived growth factor (PDGF). It also activates downstream metabolic regulators including glucose transporter 1 (GLUT1), carbonic anhydrase IX (CAIX), and erythropoietin (EPO) in a way that facilitates nutrient uptake and supports tumor cell proliferation (17–19). Moreover, HIF signaling activates pro-inflammatory cytokines like IL-6, which in turn creates a pro-tumorigenic inflammatory microenvironment (20).

Given these alterations in cellular metabolism, it is therefore not surprising that numerous prior studies have shown that obesity is a major risk factor for increased clear cell RCC incidence. Obesity also increases systemic and local inflammation and can impair tumor immunity. For these reasons, multiple groups have examined the effects of obesity on IO-based treatment outcomes in RCC and other tumor types. Conflicting results have been obtained, and currently there is no clear consensus of when or why obesity is beneficial versus detrimental in individual patients.

This review seeks to summarize current knowledge related to the metabolic effects of obesity on tumor biology and immune dysfunction in the context of RCC, with particular emphasis on the roles of adipose-tumor interactions, nutrient metabolism, and tumor immunity. While doing so, we will highlight current controversies and knowledge gaps in the field, as these will be critical areas to address in future research. Understanding interactions between host obesity, RCC biology, and tumor immunity are needed to gain a clearer understanding of RCC that can be leveraged to develop novel targeted approaches to treatment, particularly in those patients who have obesity-associated metabolic alterations. Rather than considering obesity only as a BMI-defined clinical risk factor, this review uses an immunometabolic approach to connect adipose–tumor crosstalk, lipid and glucose reprogramming, cytokine signaling, and leukocyte remodeling in RCC. We aim to explain why obesity may produce divergent clinical outcomes in individual patients by identifying the metabolic and immune states that shape tumor progression and response to ICI therapy.

## 2 Obesity as a risk factor and outcome moderator for RCC

Obesity, as measured by body mass index (BMI), is a recognized risk factor for RCC. However, as a significant proportion of data collected in epidemiological studies lacks information on RCC histotypes, risk estimates are often dominated by data on clear cell RCC (2). A systematic review of 17 studies reported a 24% increase in RCC risk in men and a 34% increase in RCC risk in women per every 5 kg/m² increase in BMI (21). A pooled analysis of 24 studies including more than 8 million people showed a 5% increase in RCC risk in men and a 6% increase in RCC risk in women per 1 kg/m² increase in BMI (22). A longitudinal cohort study in women showed that the association between BMI and RCC risk remained significant when adjusted for age; each 1 kg/m² increase in BMI correlated with a 3% increase in RCC risk (23). It is worth noting that risk factor association is not limited to BMI; in a longitudinal cohort study in women, a 0.1 unit increase in waist-to-hip ratio correlated with a 24% increase in RCC risk (23). General obesity, defined by a BMI of 25 or higher, was related to a 32% increased risk of RCC in a population of 23 million adults in Korea (24). The same dataset revealed that every increase of 5 centimeters in waist circumference was related to a 12.5% increase in RCC risk. Radiological data have also confirmed that RCC risk is related to increased levels of visceral adipose tissue (25). However, there has been some concern raised over the use of height normalization in these data (26).

The duration of exposure to excessive weight also seems to have some impact on risk, with cumulative average BMI related to increased risk for both RCC incidence and RCC fatalities (27).Weight fluctuations also significantly impact RCC incidence; adults experiencing 10 or more cycles of weight loss and subsequent weight gain of 10 pounds or more during a 7.7-year period were more than twice as likely to develop RCC compared with people without weight fluctuations (23). Public health data from the International Agency for Research on Cancer (IARC) have confirmed that prevention of weight gain has a protective impact on kidney cancer risk (28). Data from the Me-Can study, which included over 560, 000 adults, revealed that increased levels of BMI, blood pressure, glucose, and triglycerides have all been related to increased RCC risk in men. However, in women, elevated BMI was found to have the strongest association with RCC risk (29).

Notably, obesity is not only a risk factor for developing RCC but is associated with a parallel reduction in anti-tumor immunity, which dictates RCC progression, especially in the setting of immunotherapy. Indeed, experimental studies have provided evidence of a role for obesity in lymph node atrophy, altered lymphatic flow, and reduced T cell receptor diversity, which are key determinants of anti-tumor immunity (30, 31). Within tumors, diet-induced obesity in mouse models has been linked to enhanced renal tumor progression and impaired antitumor immunity, including increased MDSC accumulation, altered dendritic-cell function, reduced effector CD8^+^ T-cell activity, and diminished response to immune checkpoint blockade (32, 33). However, a series of retrospective studies in RCC patients provide a layer of complexity to these experimental studies, in which, despite a clear role for obesity in increasing RCC risk, some studies have reported improved outcomes in patients with obesity who receive systemic IO therapies; this phenomenon is referred to as the obesity paradox (34–36). Although studies in support of the obesity paradox are common, it is not a universal finding in the RCC literature, and the presence of obesity does not correspond to a positive outcome in every patient.

Indeed, human RCC studies suggest that obesity is associated with selective and patient-specific changes rather than uniform immune suppression, including reductions in some circulating leukocyte populations, relative stability of many intratumoral immune features, increased intratumoral VEGF-A/PLGF in treatment-naive ccRCC, and, in anti-PD-1-treated disease, greater IL-1β-associated inflammation, increased MDSC accumulation, and worse clinical outcomes (37–40). Overall, it is the net effect of many obesity-related factors – including the location of fat depots, the amount of systemic inflammation present, the degree of metabolic dysfunction within tumors, and the nature of the intratumoral immune response - that dictate responses in individual patients. The obesity paradox in RCC and beneficial versus detrimental effects of obesity on tumor metabolism and immunity will be discussed in more detail in subsequent sections.

## 3 Adipose tissue as an immunometabolic organ shaping renal cancer in obesity

**Figure 1:**
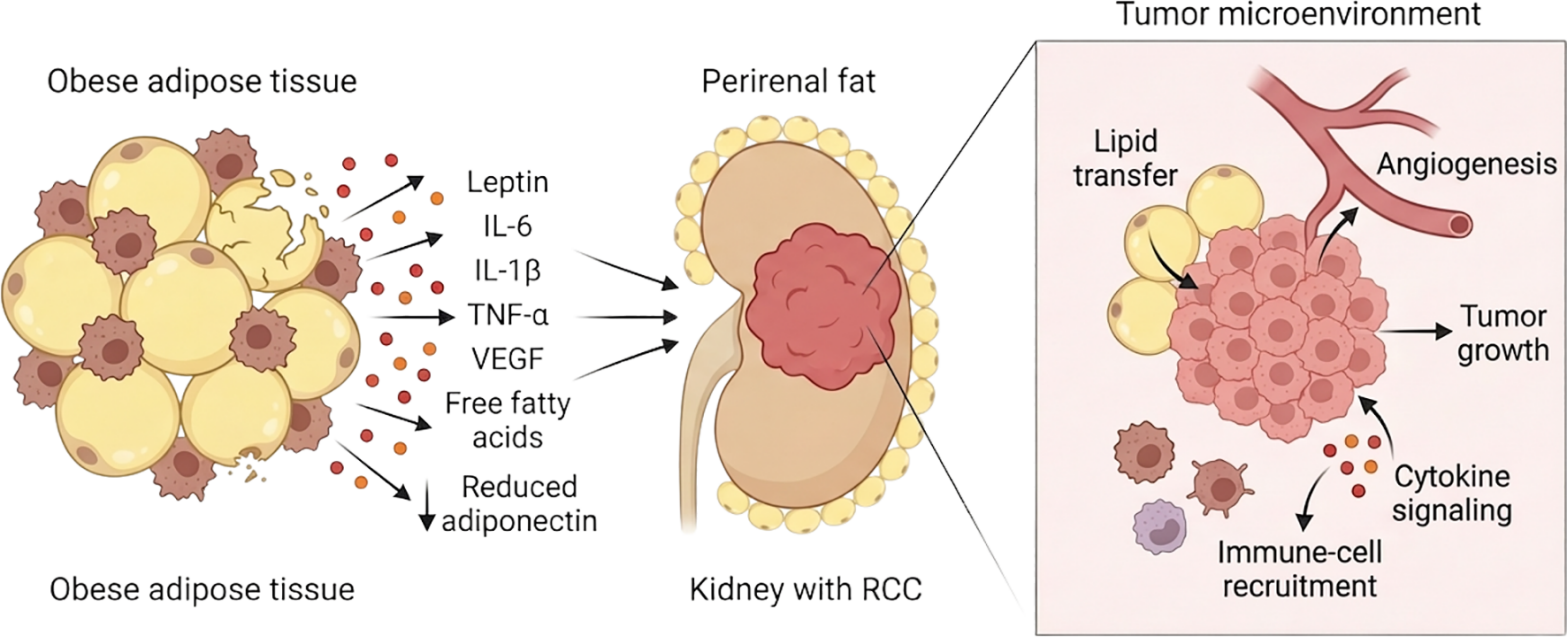
Obesity-driven adipose–kidney–tumor crosstalk in RCC. Obesity is characterized by adipose tissue release of adipokines, cytokines, angiogenic factors, and fatty acids, including increases in leptin, IL-6, IL-1β, TNFα, VEGF, and reductions in adiponectin. These mediators are secreted by perirenal fat depots to regulate the RCC tumor microenvironment, resulting in increased lipid transfer, proinflammatory cytokine signaling, recruitment of macrophages and other myeloid cells, variable T cell infiltration depending on cohort and treatment context, and enhanced angiogenesis. The arrows indicate the release of obesity-driven adipose signaling toward tumor metabolic adaptation, immune-cell recruitment, angiogenesis, and tumor growth. Figure prepared using BioRender.com.

Obesity is characterized by abnormal increases in adiposity, most typically in white adipose tissue in visceral and subcutaneous depots. Adipose tissue is an organ that is not only hormonally active but also immunologically active, indicating roles beyond that of an inactive reservoir for the storage of lipids. Adipose tissues produce various bioactive molecules such as adipokines, proinflammatory cytokines, and angiogenic factors such as TNF-α, IL-6, and VEGF (**Figure 1**) (41, 42). The bioactive molecules secreted by adipose tissues rely upon the complex composition of white adipose tissue, which consists of adipocytes plus various populations of leukocytes. Obesity is often associated with chronic nutrient overload that results in adipocyte hypertrophy and hyperplasia. This leads to the expansion of visceral fat pads and results in increased secretion of inflammatory cytokines (**Figure 1**) (41). Hypertrophic adipocytes secrete high amounts of circulating free fatty acids, which are then absorbed by insulin-responsive tissues, contributing to systemic insulin resistance and metabolic stress (43). Malignant cell growth is energy-intensive; thus meeting the continuous metabolic demands of cancerous cells is essential for tumor promotion (44). In RCC, these changes take on significance due to the proximity of renal tumors to metabolically active adipose tissues. Perirenal and peritumoral fat are active components of the renal tumor niche rather than passive bystanders; for example, adipocytes and stromal cells from these depots can release IL-6, leptin, VEGF, free fatty acids, and matrix-remodeling mediators that enhance RCC cell proliferation, migration, angiogenesis, and inflammatory signaling within nearby renal tissues (45–47). Adipocytes within the tumor microenvironment can further provide lipid substrates and paracrine signals that support tumor cell metabolism, endothelial activation, and recruitment or polarization of immune cells; they also release proangiogenic factors that influence RCC progression (48–50).

Excess adiposity generates a local environment that favors tumor growth and metastasis, not only because of the support provided to tumor growth in terms of energy supply, but also because of the continuous inflammatory input (44). Within such a local environment, signaling pathways associated with obesity can maintain a chronic inflammatory state that favors tumor growth. For instance, TNF-α can promote survival through activation of NF-κB–dependent pathways, whereas IL-6 can signal through JAK2 and PI3K/AKT cascades to stimulate cancer cell proliferation and survival. These inflammatory networks are relevant to RCC, where TNF-α–mediated pathways are also active and contribute to immune suppression by interfering with T cell function and weakening adaptive immune responses (51–53). As inflammation persists during adipose–tumor cell interactions, adipocytes can undergo phenotypic remodeling characterized by lipid depletion, increased secretion of inflammatory mediators such as IL-6 and IL-8, and acquisition of fibroblast-like features that support tumor growth (54). Expansion of the adipose stromal compartment in obesity further enhances angiogenesis and facilitates metastatic spread, as reported in other obesity–cancer models (54–56). Evidence indicates that peritumoral adipose tissue in RCC patients with obesity exhibits higher infiltration by macrophages, CD8^+^ T cells, and other leukocyte populations, together with hypoxia, with the strongest signals closest to the tumor border, supporting a model in which local fat may shape immune cell availability in the immediate tumor microenvironment and potentially act as an immune cell reservoir during immune checkpoint therapy (**Figure 1**) (34, 57, 58). These findings suggest that not only does adipose tissue surround renal tumors, but it may also play a role in shaping the RCC tumor microenvironment. More recent clinical evidence suggests that metabolic syndrome, which is characterized by high abdominal obesity among other factors, may be associated with more aggressive RCC features, including larger tumor size, higher Fuhrman grade, and more advanced tumor stage (59). Collectively, these findings support a model in which the location and type of fat depots (ex: visceral versus peri-renal vs subcutaneous) present in an individual may dictate the extent to which obesity promotes RCC progression and treatment resistance. The next section will discuss the role of the obesity-associated adipokines in RCC and how these adipokines promote RCC development and its associated complications.

## 4 Obesity-associated adipokines in RCC

### 4.1 Adiponectin

Adiponectin is an insulin-sensitizing adipokine that regulates glucose and lipid homeostasis and is primarily secreted by white adipose tissue. Its circulating levels are shaped by diet, physical activity, and abdominal adiposity, as well as tumor growth (60, 61). Obesity is characterized by reduced serum adiponectin (**Figure 1**), whereas weight loss raises adiponectin concentrations (62). Adiponectin signals via AdipoR1 and AdipoR2 and binds T-cadherin, illustrating a receptor network through which it can act on tumors (63). Experimental evidence from non-RCC cancer models suggests that adiponectin can exert anti-proliferative and pro-apoptotic effects on cancer cells (64–66). Adiponectin also suppresses mTOR and STAT3 pathways while enhancing AMPK and caspase activity, changes that impair angiogenesis and macrophage infiltration. It has further been reported to disrupt PI3K/AKT, ERK1/2, STAT3, and WNT/β-catenin signaling, causing cell-cycle arrest and apoptosis and promoting anti-tumor effects (64). Epidemiologic data consistently connect lower adiponectin with higher RCC incidence, and one report found a reduced RCC risk per each one–standard deviation increase in adiponectin (67, 68). Low circulating adiponectin has also been linked to larger RCC tumor size and metastasis, with lower circulating concentrations found in metastatic than non-metastatic RCC (69).

### 4.2 Leptin

In contrast to adiponectin, leptin is an adipokine whose circulating concentration is elevated with obesity (**Figure 1**). In human studies, obesity-associated increases in leptin have been linked to a higher overall prevalence of cancer (70–72). This is likely because sustained leptin exposure supports tumor growth through effects on stroma and blood vessels (71). This idea is supported by findings that leptin enhances VEGF expression through activation of HIF1 and NF-κB pathways, thereby promoting angiogenesis and sustaining renal tumor growth (71). Leptin Receptor (LEPR) signaling also activates downstream PI3K/AKT, MAPK, and JAK/STAT pathways that support tumor cell proliferation and survival (72, 73). In the Caki-2 RCC cell line, leptin increased proliferation and reduced apoptosis via ERK1/2 and JAK/STAT3 signaling (74). Leptin also promotes angiogenic programming and EMT-like changes that can facilitate tumor invasion and metastasis (71). In RCC, increased levels of circulating leptin and LEPR expression have been linked to more invasive tumor phenotypes and rapid tumor progression (75). In a ccRCC cohort, higher leptin concentrations were associated with poorer overall survival, and pathway analysis showed enrichment of EMT and migration programs in the high leptin group (76). Leptin also intersects with immune dysfunction in obesity-associated RCC. In treatment-naïve RCC patients, higher leptin was associated with fewer PD-1^+^ CD8^+^ T cells in blood, and higher BMI in the same cohort was linked to reduced frequencies of activated PD-1^+^ CD8^+^ T cells in tumors (40). In contrast, Khojandi et al. reported that in a cohort of mixed melanoma and breast cancer patients, plasma leptin and BMI did not correlate with higher PD-1 expression on peripheral CD8^+^ T cells (77). Thus, there may be tumor-specific effects of leptin on T cell immunity, an idea that should be pursued further.

### 4.3 Omentin-1 (intelectin-1)

Omentin-1, or intelectin-1, is an adipokine secreted preferentially by visceral rather than subcutaneous adipose tissue that acts primarily as an insulin-sensitizing and anti-inflammatory mediator, enhancing insulin signaling and suppressing inflammatory pathway activation (78). In obesity-linked metabolic disease, circulating omentin-1 levels are reduced, indicating diminished availability of an adipokine with anti-inflammatory effects. In tumors, omentin-1 is described as tumor-suppressive and pro-apoptotic, with anti-inflammatory and antioxidant effects that include p53 upregulation (79, 80). In RCC, plasma omentin levels are reduced in RCC patients versus healthy controls, and these are decreased further in RCC patients as BMI increases (81, 82). Thus, omentin-1 reductions with obesity would be predicted to promote RCC survival and progression. However, this idea is not currently supported by mechanistic data, indicating this as an area for future study.

### 4.4 Resistin

Resistin is a pro-inflammatory adipokine associated with obesity, insulin resistance, and chronic metabolic inflammation. In obesity, persistent inflammation and altered adipokine output raise circulating resistin concentrations (83). Within tumors, resistin promotes proliferation, inflammatory signaling, anti-apoptotic programs, angiogenesis, and metastatic growth. These effects are linked to MAPK activation, increased chemokine production, and increased matrix metalloproteinase expression (84). Resistin has been reported to signal through a TLR4-linked receptor pathway, activating NF-κB in macrophages via stress kinases such as JNK and p38 MAPK (85). The net results are increases in inflammatory gene expression and support of a pro-tumor inflammatory state. In RCC, circulating resistin levels have been reported to be higher in patients with advanced-stage disease than in those with localized tumors, and persistently elevated or rising postoperative resistin during follow-up has been associated with increased risk of recurrence, disease progression, and poorer cancer-specific survival (86).

Together, these findings indicated that obesity changes the balance of adipokines in and around renal tumors. Protective signals such as adiponectin and omentin-1 tend to decrease, whereas tumor-promoting signals such as leptin and resistin tend to increase. In RCC, leptin and resistin are closely linked with inflammation, angiogenesis, immune dysfunction, and disease progression. Among these markers, the roles of leptin and adiponectin in RCC are supported by robust evidence, but omentin-1 as a potential regulator of RCC progression and therapy response in the context of obesity remains an area for investigation.

## 5 Lipid reprogramming in RCC

Obesity modulates lipid availability by enhancing fatty acid release from adipose tissue, thereby altering systemic lipid metabolism and generating a lipid-rich microenvironment susceptible to tumor exploitation. The accumulation of excess lipids in cancer cells is not just a substrate supply problem, but instead acts as an organizing upstream signal that connects obesity at the systemic level with metabolic changes, inflammation, and immunosuppression within renal tumors. In RCC, these obesity-related alterations may reinforce a lipid-reprogrammed state, marked by augmented lipid uptake, *de novo* synthesis, and intracellular storage. Consequently, renal tumors may develop a reliance on lipids and fatty acid metabolism to meet energy demands and membrane architecture (87).

**Figure 2:**
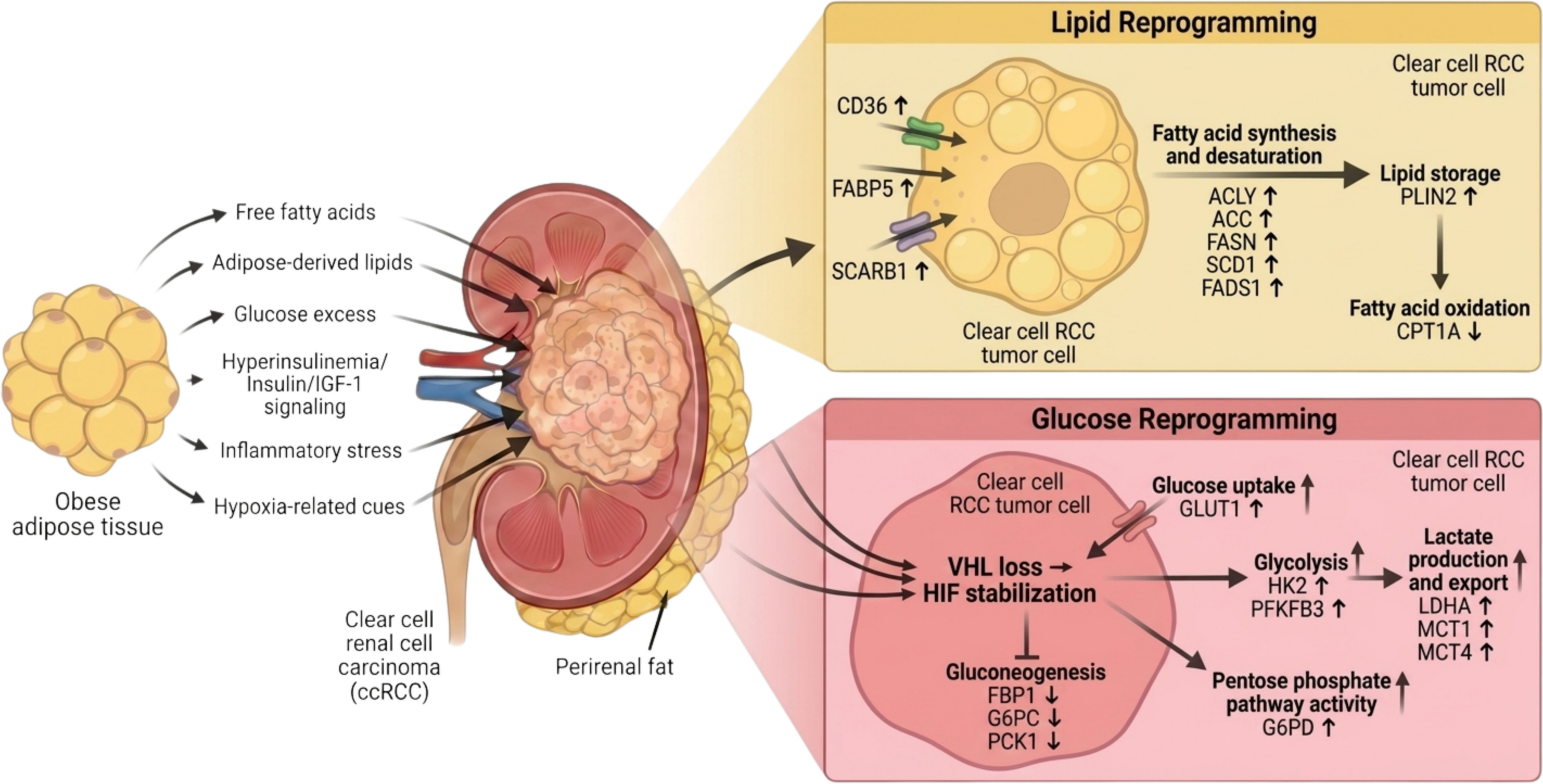
Obesity-linked metabolic reprogramming in clear cell RCC. Obesity may reinforce metabolic rewiring in clear cell RCC through increased free fatty acids, adipose-derived lipids, glucose excess, hyperinsulinemia/insulin–IGF-1 signaling, inflammatory stress, and hypoxia-related cues. In lipid metabolism, tumor cells show increased lipid uptake, synthesis, desaturation, and storage, with reduced fatty acid oxidation. In glucose metabolism, VHL loss and HIF stabilization promote increased glucose uptake, glycolysis, pentose phosphate pathway activity, and lactate production/export, together with suppression of gluconeogenesis. These metabolic arrows summarize how obesity-associated nutrient excess and hypoxia may strengthen lipid accumulation, glycolytic adaptation, and tumor survival pathways in ccRCC. Figure created with BioRender.com.

In clear cell RCC, lipid pathways are reorganized to favor increased lipid uptake, *de novo* synthesis, and intracellular storage, accompanied by diminished fatty acid oxidation. This disequilibrium results in the accumulation of fatty acids, triglycerides, cholesterol, unsaturated lipids, and abundant cytoplasmic lipid droplets within tumor cells (**Figure 2**) (88). These lipids function not as passive byproducts but as active metabolic and structural substrates that support membrane synthesis and remodeling, intracellular signaling, cellular proliferation, and adaptation to fluctuating oxygen and nutrient availability. Consequently, lipid enrichment constitutes a central component of tumor survival programs rather than a mere consequence of excessive dietary intake (89). This lipid-enriched state may also affect leukocyte composition and function in the TME through the selection of lipid-enriched macrophages, myeloid-derived suppressor cells, dysfunctional dendritic cells, and inhibition of anti-tumor T cells (89, 90). Lipid reprogramming is, therefore, an intermediary pathway linking obesity-induced metabolic stress to immune malfunction in RCC.

ccRCC tumors exhibit an accumulation of stored lipids enriched in oleate (18:1), which supports membrane remodeling and adaptation to cellular stress (91). Stearoyl-CoA desaturase-1 (SCD1) facilitates the conversion of saturated fatty acids to monounsaturated fatty acids and represents a functional dependency in ccRCC, with SCD1 activity promoting tumor growth and viability and associations with adverse clinicopathologic features in patient datasets (92). Polyunsaturated fatty acids (PUFAs) introduce additional complexity, serving as both membrane constituents and precursors for lipid mediators. In preclinical renal cancer models, PUFA desaturation governed by fatty acid desaturase 1 (FADS1) promotes RCC cell survival under stress, and inhibition of FADS1 suppresses RCC growth (93–95). PUFA enrichment generates a metabolic vulnerability due to the high susceptibility of free PUFAs to oxidation and lipid peroxidation (95). ccRCC cells counteract this oxidation by sequestering excess fatty acids within lipid droplets, which function as protective storage sites, thereby buffering oxidative stress while maintaining a lipid reservoir for future biosynthetic demands of cancer cells. This adaptive storage strategy provides a mechanistic explanation for the conserved histologic presence of lipid droplets in clear cell RCC (89, 94), as it may impart a survival advantage on these cells.

## 6 Biomarkers in lipid metabolism in RCC

Obesity is frequently accompanied by hyperlipidemia, resulting in increased lipid availability and altered lipid metabolism in ways that reinforce the lipid-rich phenotype characteristic of RCC. Consequently, lipid metabolism biomarkers aid in delineating how tumors acquire, store, and utilize lipids. In RCC, these biomarkers encompass fatty acid uptake systems, intracellular lipid chaperones, enzymes governing lipid synthesis and desaturation, regulators of fatty acid oxidation, lipid droplet structural proteins, and nodes involved in cholesterol uptake and synthesis. Collectively, these markers map the trajectory of lipids from acquisition through utilization to storage within the tumor cells (16). Given that clear cell RCC is defined by both enhanced lipid accumulation and altered lipid utilization, biomarker panels integrating uptake, storage, and oxidation yield a more accurate portrayal of disease biology than single-pathway readouts (91). Among uptake markers, CD36, a principal fatty acid translocase, exhibits strong expression in clear cell RCC and correlates with more advanced disease stage, greater visceral adiposity, and shortened progression-free and overall survival (**Figure 2**). Higher levels of CD36 have been observed to correlate positively with the body mass index and proportion of visceral adipose tissue, but negatively correlated with the proportion of subcutaneous adipose tissue. RCC tumors with higher CD36 expression appear to have more intracellular lipid accumulation and greater infiltration by CD163^+^ macrophages, with a possible reduction in intratumoral CD8^+^ T cells. This suggests that lipid uptake in ccRCC may be linked to a more macrophage-rich and less T cell-active tumor environment (96).

Within cells, fatty acid binding proteins (FABP) are lipid chaperones that control whether fatty acids are stored or utilized for metabolic purposes. Prior research has shown that the FABP5 protein is responsible for the development of obesity through its influence on diet-induced weight gain (97). Thus, in studies conducted on mice fed with a high-fat diet, the absence of FABP5 resulted in reduced body weight gain and fat mass accumulation, indicating involvement of this protein in the development of obesity (97). In clear cell RCC, the levels of FABP5 are high, correlating positively with cancer progression and metastasis, as well as poor prognosis. The knockdown of FABP5 leads to inhibition of proliferation and clonogenic growth, as well as suppression of epithelial-to-mesenchymal transition, along with lower levels of PI3K and AKT signaling (98). Importantly, FABP5 can also be considered a biomarker for ccRCC, since the levels of this protein are high in the serum and urine samples of affected individuals (99).

Enzymes that drive lipid synthesis form another important biomarker class. In obesity, increased activity of lipogenic enzymes such as ATP citrate lyase (ACLY) and acetyl CoA carboxylase (ACC) support *de novo* fatty acid synthesis and triglyceride accumulation in metabolic tissues (**Figure 2**) (100). ACLY, which generates acetyl CoA from citrate, is consistently overexpressed in clear cell RCC in both transcriptomic and proteomic datasets and correlates with poorer overall and progression-free survival (101). Acetyl CoA carboxylase (ACC), the rate limiting enzyme in *de novo* lipogenesis, is likewise linked to adverse prognosis and reduced survival (102). Together, high ACLY and ACC, along with suppression of AMPK, which normally restrains lipid synthesis, define a biomarker axis of enhanced lipogenesis and energy imbalance in clear cell RCC (101, 103). Fatty acid synthase (FASN), an enzyme involved in fatty acid production, is an important lipid biomarker in RCC and provided early clues linking lipid metabolism to the obesity paradox in metastatic disease. Albiges et al. established an association between higher BMI (> 25 kg/m^2^), low FASN expression, and better overall survival in RCC patients treated with targeted therapies; in lean patients, increased FASN expression was linked to poor survival and heightened tumor aggressiveness (104). Desaturation of fatty acids can be measured through stearoyl-CoA desaturase 1 (SCD1) – the enzyme responsible for saturation transformation from saturated to monounsaturated fatty acids (92, 105). SCD1 signaling is typically elevated with obesity. SCD1 is also frequently overexpressed in clear cell RCCs, where it contributes to tumor cell survival by activating the PI3K/AKT signaling pathway and increasing lipid droplet abundance – ultimately leading to poor prognosis. Moreover, it is suggested that SCD1 can serve as a marker not only of dependence but also of imaging and response prediction in treatment and thus as a therapeutic target (105). Fatty acid remodeling can be estimated by assessing fatty acid desaturase 1 (FADS1) – the enzyme that regulates fatty acid desaturation and is responsible for modifying membranes, inflammation, and peroxidation. FADS1 is commonly overexpressed in clear cell RCC, especially when high-grade tumors contain large amounts of PUFA-containing phospholipids. Despite the fact that PUFA levels are decreased even at high FADS1 expression, such data indicate preferential integration of these compounds into membranes, which means that FADS1 serves as a marker of fatty acid remodeling but not as a measure of total lipids (106).

Clear cell RCC cells are characterized by a reduced ability to oxidize fatty acids, and there are many different biomarkers that reflect this process. It is unknown whether obesity is associated with similar effects, but metabolic stress caused by obesity may promote fat storage rather than its oxidation. One of the enzymes that transport fatty acids to mitochondria, where they are oxidized, is CPT1A. With increasing HIF activation due to the lack of VHL, CPT1A expression is inhibited, promoting intracellular lipid accumulation (87). CPT1A recovery leads to decreased lipid storage and slower tumor growth making it a potential prognostic and therapeutic target (87). Enoyl-CoA hydratase short chain 1 (ECHS1) performs fatty acid oxidation within the mitochondria. Its expression is reduced with advancing RCC stage and it can be used as a distinguishing factor between tumor and healthy kidney tissue. With increased metabolic stress and reduced mitochondrial fatty acid oxidation that accompany obesity, one might predict lower ECHS1 expression or activity, consistent with impaired fatty acid oxidation; however, this relationship has not been directly established in obesity-associated RCC. However, in ccRCC ECHS1 is overexpressed and leads to mTOR suppression, thereby inhibiting cancer cell proliferation and migration (107). In clear-cell RCC, PLIN2/adipophilin marks lipid droplets and promotes HIF2α-dependent lipid storage that supports tumor-cell viability, while high membranous adipophilin expression has been linked to poorer survival in RCC patients (108, 109). In obesity-associated RCC, increased lipid availability may further favor PLIN2-linked lipid storage, although direct evidence for this remains limited. Clinically, these markers could help show which RCC tumors rely more heavily on lipid uptake, lipid storage, or reduced fatty acid oxidation. This may help predict prognosis, group patients more accurately, and guide future treatments that specifically target lipid metabolism in obesity-associated RCC.

## 7 Glucose metabolic reprogramming in RCC

In addition to perturbations in lipid metabolism, RCC demonstrates significant alterations in glucose metabolism. These adaptations support tumor growth and adaptation to stressful environmental conditions. In many adults with obesity, particularly those with insulin resistance, impaired glucose tolerance, or type 2 diabetes, systemic glucose levels may be elevated, which can promote development of an inflammatory and hypoxic environment that facilitates tumor glycolysis (41). The distinguishing feature of ccRCC involves its preference for aerobic glycolysis, which includes the diversion of glucose to anabolic processes, rather than oxidative processes in mitochondria (110). Characteristic features include increased pentose phosphate pathway (PPP) activity and reduced expression of tricarboxylic acid (TCA) cycle and oxidative phosphorylation genes, all of which support tumors that need rapid access to ATP and biosynthetic precursors under variable oxygen and nutrient conditions (102). Li et al. found that ccRCC also suppresses key gluconeogenic enzymes, including glucose 6 phosphatase (G6PC), fructose 1, 6 bisphosphatase 1 (FBP1), and phosphoenolpyruvate carboxykinase 1 (PCK1), which keep cells in a glucose expenditure state (111). Metabolite analyses confirm enhanced glucose uptake and utilization in tumor tissue, so this shift is evident at both transcriptional and metabolite levels (112). In human ccRCC, isotope tracing has shown higher glycolytic flux than in adjacent normal kidney tissue together with reduced pyruvate dehydrogenase flux, reduced TCA cycle activity, and lower glucose oxidation, confirming that glycolysis is directly increased in RCC (113). In primary ex vivo ccRCC tissues and RCC cell models, this glycolytic state is also functionally important, because inhibition of glycolysis impairs ccRCC viability and proliferation (114). In obesity, chronic hyperinsulinemia, increased glucose availability, and inflammatory and hypoxic signaling would be expected to further favor glycolytic metabolism. However, direct evidence that these glycolytic pathways are specifically increased in RCC tumors from patients with obesity compared with those without obesity remains limited. Thus, in obesity-associated RCC, it appears that obesity may reinforce an already established glycolytic program in ccRCC.

This glucose predominant phenotype is closely linked to aberrant oxygen sensing and hypoxia responses in ccRCC. Loss of VHL, a defining hallmark of most ccRCC, increases glucose uptake through stabilization of hypoxia-inducible factors, which upregulate glucose transporter 1 (GLUT1) and reinforce glycolytic metabolism (**Figure 2**) (17, 20). Wolf et al. demonstrated that in VHL-deficient ccRCC tumors, tumor-associated macrophages show enhanced glucose uptake, greater phagocytic activity, and pro-inflammatory transcriptional programs (115). T cells in this setting have reduced effector cytokine secretion and show poor responses to PD-1 blockade, consistent with metabolic competition as one mechanism of immunotherapy resistance (115). Glucose metabolism in cancer cells is controlled at entry, rate limiting steps, and endpoint conversion rather than as a simple on or off switch. In RCC, oncogenic signaling increases glucose transporter (GLUT) activity to enhance glucose uptake. One described pathway shows S100A2 binding hepatocyte nuclear factor 1 homeobox A (HNF1A) to drive GLUT2 expression, which increases glucose uptake, glycolytic activity, and aggressive tumor behavior (116). ccRCC also shows high glycolytic flux, with more glucose entering the pentose phosphate pathway (PPP) (117). Intermediate products of the PPP are higher, and there is a positive correlation between glucose-6-phosphate dehydrogenase (G6PDH) activity and intermediate accumulation. Inhibiting the action of G6PDH leads to decreased cell survival, lower concentrations of NADPH, and an increase in ROS generation, suggesting that PPP is involved in maintaining cellular redox balance in ccRCC (112). Thus, the available information proves that glucose metabolism has a significant role in ccRCC development and progression. This makes it important to examine the biomarkers that best reflect glucose metabolic reprogramming in RCC.

## 8 Glucose metabolism biomarkers in RCC

Obesity alters systemic metabolism in ways that may support the glycolytic phenotype of ccRCC. Increased glucose availability, higher lactate production, and adipose metabolic dysfunction create conditions that favor tumor glucose use (114). In ccRCC, glucose metabolism biomarkers include glucose transporters, glycolytic enzymes, lactate transporters, pentose phosphate pathway enzymes, and gluconeogenic enzymes. Together, these markers reflect a coordinated shift toward increased glycolysis, greater lactate production, and reduced oxidative metabolism (13). In obesity, enlarged adipocytes increase basal glucose uptake, and higher glucose transporter 1 (GLUT1) expression contributes to greater glycolytic flux and lactate release, promoting inflammation and insulin resistance (**Figure 2**) (118). In ccRCC, loss of von Hippel-Lindau (VHL) stabilizes hypoxia-inducible factor 1 alpha (HIF-1α) and strongly induces GLUT1 expression, sustaining high glucose uptake in tumor cells. Consequently, glucose influx into ccRCC through obesity-mediated hyperglycemia and enhanced expression of GLUT1 in tumor could work together to increase glucose influx into cancerous tissues and to promote dependence on glycolysis (110).

The early regulation of glycolysis is also affected by hexokinases. Obesity induces downregulation of hexokinase 2 (HK2) in adipose tissue, reducing glucose utilization and insulin-stimulated glucose handling, thereby contributing to hyperglycemia and insulin resistance (119). In contrast, upregulation of HK2 and its association with enhanced glycolysis, poor prognosis, higher tumor stages, lymph node metastasis, and low overall survival rates have been reported in ccRCC (120). In human clear-cell RCC, HK3 is upregulated and is associated with poor survival, increased monocyte/macrophage infiltration, and higher PD-1 and CTLA-4 expression (121). Thus, while obesity impairs glucose uptake peripherally, ccRCC increases glucose consumption by tumors; hence, excess glucose becomes available for tumor metabolism.

PFKFB3 is reduced in obese adipose tissue, which leads to impaired adipocyte glycolysis, adipose inflammation, and glucose intolerance (122). In contrast, PFKFB3 is increased in human RCC/ccRCC, where it promotes glycolysis, tumor-cell proliferation, advanced TNM stage, and poor prognosis (123). In adipose tissue, PGAM1 regulates glycolytic metabolism and adipose remodeling, while in human clear-cell RCC, PGAM1 is overexpressed and is associated with larger tumor size and higher T stage (124, 125). Together these changes contribute to elevated glucose levels with obesity, allowing RCC to capitalize on PFKFB3/PGAM1-mediated pathways to support anabolism.

During glycolysis, the metabolism of lactate becomes even more important. Individuals with obesity produce excess lactate through the actions of lactate dehydrogenase A (LDHA), which results in the activation of macrophages and inflammation (118). In RCC, the level of LDHA is highly elevated, and it contributes to the Warburg effect, which is the transformation of glucdcose into lactate in the presence of oxygen. Increased levels of LDHA correspond to larger ccRCC tumors, higher grades, epithelial-mesenchymal transition, and metastasis (126). Hypoxic human adipocytes increase monocarboxylate transporter 1 (MCT) and monocarboxylate transporter 4 (MCT4) expression to support lactate export during adipose dysfunction (127). In human ccRCC, high MCT1 or MCT4 expression is associated with reduced overall survival and progression-free survival (128).

In general, current literature suggests that obesity can intensify the glycolytic phenotypic behavior of RCC through increased levels of glucose and lactate in the systemic circulation while suppressing glucose and oxidative metabolism in peripheral insulin-responsive tissues such as adipose tissue and skeletal muscle. RCC tumors take advantage of this microenvironment by activating several factors including GLUT1, HK2, HK3, PFKFB3, PGAM1, LDHA, MCT1, and MCT4 to facilitate the glycolytic process and enhance progression. Metabolic switching not only represents an intratumoral characteristic but also plays a significant role in the immune microenvironment of RCC, which is discussed in subsequent sections. In the clinic, glucose-related markers may help distinguish RCC tumors that are metabolically active and more dependent on glycolysis. High expression of GLUT1, HK2, PFKFB3, LDHA, MCT1, or MCT4 may point to tumors with aggressive glucose use, increased lactate handling, and a more suppressive metabolic environment. These markers could therefore help identify patients with poorer prognosis and support future strategies that target tumor glycolysis or lactate-mediated immune suppression in obesity-associated RCC.

## 9 Cytokines in obesity-associated RCC

Obesity creates a chronic inflammatory state that begins in adipose tissue, where adipocyte stress drives persistent myeloid cell activation and sustained local and systemic cytokine release. This ongoing low-grade inflammation can affect distant tissues, creating conditions that facilitate subsequent tumor growth (129). Once tumors form, cytokines further shape the microenvironment by regulating leukocyte recruitment, endothelial activation, stromal remodeling, and survival signaling in tumor cells. In RCC, cytokine networks are closely tied to hypoxia pathways, angiogenesis, myeloid skewing, and treatment response (130). They act as molecular links between immune cells, blood vessels, and tumor cells and help determine whether immune infiltration becomes cytotoxic and tumor-rejecting or shifts toward immunosuppressive tolerance. Below we discuss key obesity-related cytokines and their effects on RCC progression and outcomes.

### 9.1 Interleukin-1β

IL-1β is a classic pro-inflammatory cytokine. With obesity, IL-1β rises in response to metabolic and adipose tissue stress. Expanding fat depots recruit and activate myeloid cells, which sustain chronic IL-1β-driven inflammation (131). We reported previously that circulating IL-1β is significantly increased in RCC patients with obesity relative to tumor-free adults with obesity, circulating IL-1β levels were equivalently high in RCC patients -/+ obesity (132). Within tumors, IL-1β supports myeloid recruitment and expansion and drives angiogenic and immunosuppressive programs through downstream cytokines and chemokines. In ccRCC, Najjar et al. found evidence that IL-1β-driven MDSC expansion was associated with higher IL-8, CXCL5, and CCL3, chemokines that can enhance recruitment of neutrophils and suppressive myeloid cells, amplify inflammatory trafficking, and reinforce a protumor microenvironment associated with disease progression (133), but they did not examine these associations in the context of obesity. IL-1β also increases RCC invasiveness through a matrix metalloproteinase (MMP) program regulated by C/EBPβ, which upregulates MMP-1, MMP-3, MMP-10, and MT1-MMP (134). We examined intratumoral IL-1β in RCC patients who had obesity versus those who did not, but found no differences in our small cohort (132). However, in a preclinical model of combined obesity and orthotopic Renca renal tumor growth, we found that IL-1β was elevated in tumors from obese mice and that neutralizing IL-1β enhanced immune checkpoint inhibitor responses and inhibited intratumoral MDSC accumulation, highlighting IL-1β as a driver of obesity-related immune resistance in RCC (40). A subsequent study using the same Renca model in lean mice found that IL-1β blockade reduced polymorphonuclear myeloid-derived suppressor cell (PMN-MDSC) and TAM infiltration and improved responses to PD-1 inhibitors and cabozantinib, with fewer suppressive MDSCs and more M1-like TAMs (135). Thus, multiple lines of evidence implicate IL-1β as a key driver of myeloid cell-based immune suppression in the renal tumor microenvironment. While systemic IL-1β elevations are linked to obesity and experimental models support a functional role for intratumoral IL-1β in promoting resistance to immunotherapy, definitive evidence connecting obesity-associated IL-1β to clinical RCC outcomes in humans remains incomplete and warrants further study.

### 9.2 IL-6

IL-6 is a pro-inflammatory cytokine often elevated in obesity because inflamed adipose tissue and activated myeloid cells produce it continuously (136). In tumors, IL-6 sustains inflammatory signaling and activates survival pathways, particularly JAK/STAT3, thereby promoting tumor cell proliferation, survival, and immune dysregulation. These immune effects include support of myeloid-driven immunosuppression, impaired cytotoxic T cell activity, and reduced antitumor immune surveillance (137). In RCC, IL-6 is linked to a tumor-promoting microenvironment characterized by enhanced angiogenic signaling, immune suppression, and resistance to systemic therapy. IL-6 is part of a hypoxia-linked signaling axis: hypoxic stress can increase IL-6 through NOX4, whereas IL-6/STAT3 signaling enhances HIF-1–dependent VEGF expression and thereby promotes angiogenesis (138). Clinically, elevated IL-6 in metastatic RCC is associated with more invasive disease and poorer responses to TKIs such as sunitinib and pazopanib (139). IL-6 signaling also activates AKT, mTOR, and NF-κB, further contributing to treatment resistance. In addition, IL-6/NF-κB signaling through PAK1 has been associated with stem-like tumor phenotypes and increased sunitinib resistance (140). In VHL-deficient RCC, characterized by loss of von Hippel–Lindau function and constitutive HIF signaling, elevated IL-6 promotes macrophage recruitment and polarization toward an M2-like, pro-tumor phenotype (141). In ccRCC, IL-6/JAK/STAT3 activity impairs cytotoxic T cell activation and reduces immunotherapy efficacy (142). Thus, high IL-6 activity together with TAM enrichment may define an obesity-associated immunometabolic phenotype with greater risk of CD8^+^ T cell dysfunction and weaker response to immune checkpoint blockade.

### 9.3 IL-8

Obesity can increase IL-8/CXCL8 production through inflammatory signaling in adipose tissue and activated myeloid cells (143). In RCC, IL-8 is produced within the tumor microenvironment by macrophages and is linked to tumor-promoting effects that include enhanced RCC cell proliferation, reduced apoptosis, angiogenic signaling through VEGF/VEGFR-related pathways, and recruitment of neutrophils, tumor-associated macrophages, and myeloid-derived suppressor cells (144). IL-8 has also been associated with epithelial–mesenchymal transition, cancer stem-like features, and glycolytic reprogramming in non-RCC solid tumor models (145). Zacchi et al. found that across six advanced or metastatic RCC cohorts (n = 1762), high circulating IL-8 was associated with shorter overall survival (HR 1.85, 95% CI 1.21–2.84) and progression-free survival (HR 1.27, 95% CI 1.01–1.59). The negative association was strongest in patients treated with immunotherapy (OS HR 2.57) or everolimus (OS HR 2.01) and remained significant in TKI-treated cohorts (146). In resected ccRCC specimens, high IL-8 expression also predicted worse survival. IL-8^high^ and IL-8^low^ tumors did not separate by VHL mutation status, suggesting that IL-8 upregulation in RCC is independent of VHL (146).

### 9.4 VEGF

Obesity amplifies VEGF signaling through elevated growth factors and increased tissue hypoxia, resulting in pro-tumorigenic angiogenesis and immune suppression. Chronic hyperinsulinemia – which often accompanies obesity - increases IGF-1 activity, which signals through insulin and IGF-1 receptors to activate PI3K/AKT and drive HIF1A-dependent VEGF expression (147). RCC is highly angiogenesis-dependent because von Hippel-Lindau (VHL) loss is common and stabilizes the downstream HIF, leading to increased VEGF transcription (148). RCC reliance upon VEGF signaling explains the widespread use of VEGFR TKIs for clinical patient care. VEGF binds VEGFR2 on endothelial cells and promotes ccRCC survival and proliferation via PI3K/AKT and migration via PLCγ/PKC and Src/FAK pathways. The result is dense, disorganized vasculature that maintains hypoxia and further stimulates VEGF production (149). In metastatic RCC, VEGF pathway blockade disrupts vascular support and slows tumor growth, forming the basis for VEGFR TKIs and anti-VEGF antibodies as standard treatments (150). Beyond its role in promoting angiogenesis, VEGF perturbs immune responses by impairing dendritic cell maturation and expanding immunosuppressive myeloid cell populations (150). It is therefore not surprising that VEGF blockade - particularly in combination regimens such as bevacizumab, an anti-VEGF antibody, plus atezolizumab, an anti-PD-L1 antibody - can enhance T cell infiltration, increase MHC class I expression, and strengthen anti-tumor immunity (150, 151).

Collectively, prior studies on obesity-associated cytokines suggest that inflammation in obesity-associated RCC involves broad signaling networks that suppress protective anti-tumor immunity while promoting angiogenesis and local tissue remodeling. These cytokine-driven changes also help shape the innate immune compartment of RCC, fostering myeloid cell recruitment, polarization, and dysfunction that contribute to tumor progression.

## 10 Innate immune remodeling in RCC

Obesity promotes chronic inflammation and systemic expansion of immunosuppressive myeloid populations. In RCC, this is reflected by remodeling of MDSCs, TAMs, neutrophils, dendritic cells, and innate sensing pathways such as TLR and inflammasome signaling. Rather than acting independently, these pathways converge to create a myeloid-suppressive niche that links adipose inflammation, cytokine production, angiogenesis, and impaired antitumor immunity. The subsections below discuss each innate immune population separately, while avoiding repetition of their shared suppressive functions.

### 10.1 MDSCs

Myeloid-derived suppressor cells (MDSCs) are a heterogeneous population of immature myeloid cells that expand in cancer and inhibit T cell activation through multiple suppressive mechanisms (152). In RCC, MDSCs increase in the blood as a result of enhanced reactive myelopoiesis in the bone marrow. This results in the production of increased numbers of immature myeloid cells in the periphery known as MDSCs (133). Monocytic MDSCs (M-MDSCs) are mainly linked to immunosuppression, whereas polymorphonuclear MDSCs (PMN-MDSCs) more often support angiogenesis (153). Both subsets deplete key amino acids such as L-arginine and L-cysteine and use enzymes like arginase-1 and inducible nitric oxide synthase (iNOS) to impair T cell proliferation, JAK/STAT signaling, and survival (152, 154). TLR signaling further regulates these cells. TLR3, TLR7, TLR8, and TLR9 are associated with anti-tumor effects and lower MDSC levels, whereas TLR1 and TLR2 agonists can drive M-MDSCs toward immunosuppressive macrophages (155). A well-described mechanism involves TLR4 ligation on monocytes: soluble HSP90α binds TLR4, activates NF-κB, increases PD-L1, and reduces HLA-DR, all of which support monocyte survival and conversion into suppressive MDSCs (156). In RCC patients, higher circulating MDSC levels correlate with advanced stage, larger metastatic burden, and poorer outcomes (133). Tumor-derived IL-1β promotes PMN-MDSC accumulation, and RCC-derived exosomes rich in complement C3 induce CCL2 and CXCL1 release in lung macrophages, which helps recruit PMN-MDSCs to distant sites (157). In a murine RCC model, IL-1β promoted PMN-MDSC accumulation and suppressive myeloid remodeling; correspondingly, IL-1β blockade reduced PMN-MDSCs, reshaped the myeloid compartment, and improved response to PD-1 blockade (135). In an obesity-associated murine RCC model, Boi et al. showed that obesity intensified IL-1β-high, MDSC-rich myeloid programs and resistance to anti-PD-1 therapy, whereas IL-1β neutralization reduced intratumoral MDSC accumulation, improved checkpoint inhibitor responses, and shifted macrophages toward an M1-like phenotype (40).

### 10.2 Macrophages

Macrophages are key cells in innate remodeling in RCC and are recruited and polarized by both adipose tissue and tumor-derived signals. With obesity, adipocytes release MCP-1, IL-1β, and TGF-β, which expand and polarize monocytes toward an M2-like tumor-associated macrophage (TAM) phenotype (158). In RCC, extracellular vesicles and soluble tumor factors recruit bone marrow–derived monocytes that differentiate into TAMs at local and distant sites (159). Within tumors, innate sensing pathways activate NF-κB, STAT3, and HIF-1α, which in turn induce IL-1β, IL-6, IL-23, TNFα, and VEGFA (130). Once NF-κB is active, it increases leukocyte recruitment, angiogenesis, invasion, and resistance to apoptosis. VHL loss, a defining lesion in clear cell RCC, amplifies NF-κB activation and suppresses p53, promoting immune escape and tumor persistence (160). Inflammasome activity within macrophages further promotes immune suppression in tumors. NLRP3 complexes expressed on macrophages activate caspase-1, which processes pro–IL-1β into active IL-1β, allowing IL-1β to then expand suppressive myeloid cells (161). IL-1β further recruits TAMs and regulatory T cells and reduces cytotoxic T cell function (135). In ccRCC, high IL-1β correlates with intratumoral IL-8, CXCL5, and CCL3, which together promote further myeloid recruitment (133).

Endoplasmic reticulum (ER) stress also contributes to macrophage-mediated immune suppression. In VHL-deficient mouse kidneys, Inositol-requiring enzyme 1 alpha (IRE1α), an endoplasmic reticulum stress sensor that activates the unfolded protein response produces inflamed, fibrotic lesions with clear cell features and dense macrophage recruitment, and the same IRE1α pathway is implicated in macrophage dysfunction in obesity-related adipose disease (162), suggesting shared stress programs between obese adipose tissue and RCC. RCC cells also use epithelial–endothelial crosstalk to attract macrophages: VHL-deficient tubular epithelial cells secrete IL-6 and oncostatin M, which stimulate endothelial cells to release more IL-6 and further increase macrophage recruitment and polarization (141). In this environment, TAMs in RCC often adopt an M2-like, pro-tumor state driven in part by reduced SOX17 and exosomal long non-coding RNAs, and they promote immunosuppression and tumor growth by supporting angiogenesis through CXCL12 and other growth factors (163, 164). Bader et al. found that obesity reinforces this state, as leptin, insulin, palmitate, and pro-inflammatory cytokines induce PD-1 on TAMs through mTORC1- and glycolysis-dependent pathways, and reduce macrophage metabolic activity and phagocytosis, thereby limiting T cell activation (165). In human clear cell RCC, PD-1^+^ TAMs are detectable and distinct from TREM2^+^ lipid-associated macrophages, and high-fat diet exposure selectively expands the PD-1 macrophage pool in RCC models, indicating that obesity favors a lipid-loaded, immunosuppressive TAM population in the tumor microenvironment. In models of combined obesity and RCC, IL-1β blockade also shifts TAMs toward a more M1-like phenotype, suggesting that cytokine targeting can partially reverse obesity-associated macrophage polarization and improve antitumor immunity (135).

### 10.3 Dendritic cells

**Figure 3:**
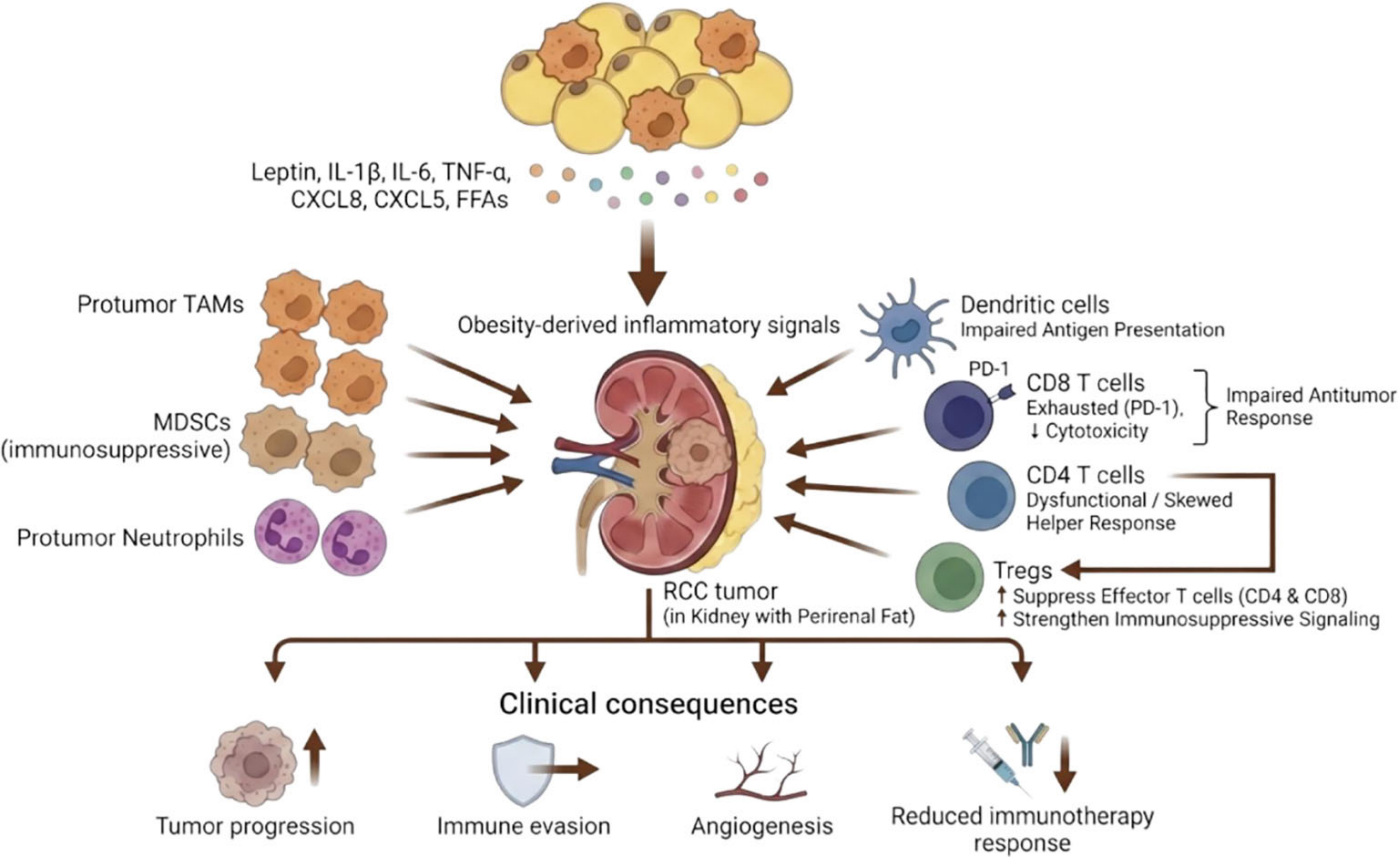
Obesity-driven immune remodeling in RCC. Obesity-associated adipose signals, including leptin, IL-1β, IL-6, TNFα, CXCL8, CXCL5, and free fatty acids, reshape the RCC microenvironment by promoting protumor TAMs, immunosuppressive MDSCs, and protumor neutrophils, while impairing dendritic-cell antigen presentation and weakening CD8^+^ and CD4^+^ T cell function. At the same time, Tregs expand and reinforce immunosuppressive signaling. Together, these changes promote tumor progression, immune evasion, angiogenesis, and reduced response to immunotherapy. Figure created with BioRender.com.

Dendritic cells (DCs) are antigen-presenting cells that capture tumor-derived antigens and process them for presentation to T cells through major histocompatibility complex class I (MHC class I) and major histocompatibility complex class II (MHC class II), thereby linking innate sensing to adaptive anti-tumor immunity (30, 166). Dendritic cells (DCs) are also altered in obesity, both systemically and in tumors. In lean adipose tissue, DCs help maintain an anti-inflammatory state and support Th2-biased responses, but obesity shifts them toward a pro-inflammatory phenotype that drives Th17 differentiation (167). In mice and humans, higher BMI correlates with more CD11c^+^CD1c^+^ DCs that can induce Th17 responses (166). Through subset-specific expression of endosomal TLRs, including TLR3, TLR7, TLR8, and TLR9, DCs sense nucleic acid-derived innate ligands and translate these signals into altered antigen presentation and T-cell priming (168). In obesity, excess lipids and adipose inflammatory signals can chronically engage TLR-associated pathways and alter myeloid-cell function toward less effective antitumor phenotypes (169). Leptin signaling further activates DCs and skews their function, whereas DCs from leptin- or leptin receptor–deficient mice show reduced co-stimulatory molecule expression and weak T cell priming (**Figure 3**) (170). We found that in mice with diet-induced obesity, Renca renal tumors were infiltrated by CD11b+ DC with decreased IL-12p70 expression that suppressed T cell proliferation (32). Thus, obesity may bais the function of tumor-infiltrating DC toward regulatory or tumor-promoting activities.

### 10.4 Neutrophils

Neutrophils also contribute to RCC progression. A high neutrophil-to-lymphocyte ratio is a negative prognostic marker in metastatic RCC, and neutrophil counts are higher in RCC patients than in healthy controls, even though absolute numbers do not clearly distinguish early from late stage disease (171–173). Neutrophil recruitment is promoted by tumor-secreted CXC chemokines that support systemic inflammation and disease progression, and expression of membrane-spanning 4-domains subfamily A (MS4A) family genes in tumor tissue has been linked to Siglec-F^high^ neutrophil infiltration, suggesting enrichment of a specialized neutrophil-associated inflammatory program (174). Because PMN-MDSCs overlap phenotypically with neutrophil-lineage suppressive cells, the neutrophil compartment may contribute to the same broader myeloid-suppressive program described above (174). In ccRCC, elevated IL-1β-associated chemokines such as IL-8, CXCL5, and CCL3 may support neutrophil-lineage recruitment (133). Obesity may amplify this interaction by increasing leptin, TNFα, CXCL1, and other inflammatory mediators that enhance neutrophil recruitment, activation, and survival. In obesity-associated RCC models, neutrophils shift toward a more immunosuppressive phenotype, with increased degranulation and higher expression of arginase-1, neutrophil elastase, and PD-L1 within the tumor microenvironment (175). Higher body mass index in adults with RCC, particularly in those with greater subcutaneous adiposity, was associated with stronger neutrophil activation, suggesting that obesity may promote an ARG1-high neutrophil state that weakens anti-tumor T cell responses and supports tumor progression (175).

## 11 Adaptive immune remodeling in RCC

Adaptive immune cells in RCC include CD8^+^ cytotoxic T cells, CD4^+^ helper T cells, and regulatory T cells (Tregs). Obesity-related chronic inflammation and metabolic dysfunction can disrupt these adaptive responses by promoting T cell exhaustion and immunosuppression (**Figure 3**). In ccRCC, effective anti-tumor control depends largely on whether tumor-infiltrating T cells remain functionally cytotoxic or instead become exhausted, whereas Treg enrichment is generally associated with worse prognosis (**Figure 3**) (176). In obesity-associated RCC, chronic exposure to leptin, inflammatory cytokines, and metabolic stress may further shift this balance away from effective CD8^+^ T cell function and toward a more suppressive adaptive immune state (71).

### 11.1 CD8 T cells

Cytotoxic CD8^+^ T cells normally control tumors by recognizing neoantigens presented on MHC class I molecules through their TCR and then releasing granzymes and perforin, which damage tumor cell membranes and cause cell death (177). ccRCC stands out among solid tumors for very high immune cytolytic activity when measured by RNA expression of granzyme A (GZMA) and perforin (PRF1), although these markers are not exclusive to CD8^+^ T cells and can also reflect contributions from cytotoxic NK cells (178). Across untreated primary tumor types, ccRCC ranks among the very highest for this cytolytic signal and also shows strong T cell and overall immune infiltration scores in TCGA-based comparisons (179, 180). Thus, ccRCC tumors are often heavily infiltrated by CD8^+^ T cells that appear, at least transcriptionally, to be armed for cytotoxic activity. However, this strong CD8^+^ entry does not translate into durable tumor control as disease progresses (180, 181). Consistent with this complexity, higher CD8A transcript abundance in bulk tumor datasets has not uniformly predicted better outcomes and in some cohorts has been associated with poorer prognosis, likely reflecting that T cell quantity alone does not capture functional state, spatial exclusion, or exhaustion (182). In early stage ccRCC, effector CD8^+^ T cells are enriched, whereas metastatic ccRCC is dominated by terminally exhausted CD8^+^ T cells (183). Earlier work also showed that ccRCC TILs are enriched for differentiated effector-memory phenotypes. This supports the idea that RCC can contain abundant tumor-reactive T cells that are present in the tumor but functionally constrained (184). This functional gap may be partially metabolic in nature, as CD8^+^ TILs from human ccRCC show impaired glucose uptake and glycolysis, fragmented hyperpolarized mitochondria, increased mitochondrial ROS, and partial restoration of activation after metabolic rescue with pyruvate or mitochondrial ROS scavengers (181). As tumor stage increases, expression of inhibitory checkpoints such as PD-1, TIM-3, and LAG-3 rises, whereas markers associated with progenitor or stem-like T cell programs, including TCF1 and T-bet, are higher in earlier disease and then decline with progression (183). TCR sequencing shows that terminally exhausted CD8^+^ T cells in ccRCC can be highly clonal and can comprise a large fraction of total intratumoral T cells, suggesting that initially tumor-reactive clones become locked in dysfunctional, exhausted states rather than being fully deleted (183).

Single-cell and mass cytometry profiling further highlight the complexity of T cell states in ccRCC. PD-1 is broadly expressed across many T cell phenotypes, while other checkpoints - such as CTLA-4, 4-1BB, and TIM-3 - show more restricted and variable patterns across CD4^+^ and CD8^+^ subsets (182). ccRCC tumors therefore contain multiple distinct CD8^+^ and CD4^+^ phenotypes, each with different checkpoint combinations, creating a mosaic of T cell states within the same tumor (185). This diversity allows for nuanced but also fragmented responses to checkpoint blockade, because not all T cell subsets share the same “exhaustion” or activation profile. Myeloid cell programs align with these T cell checkpoint patterns as disease advances. With progression, tumor-associated macrophages (TAMs) shift from more pro-inflammatory transcriptional programs toward anti-inflammatory, M2-skewed gene expression, especially in metastatic disease (182). M2-like TAMs can express multiple checkpoint ligands, including PD-L1, CD80/CD86, CD155, and Galectin-9, which engage PD-1, CTLA-4, TIGIT, and TIM-3 on T cells (186). These macrophage-T cell ligand interactions are associated with worse overall survival in ccRCC (183). Thus, CD8^+^ dysfunction in advanced RCC reflects not only intrinsic T cell exhaustion but also an immunosuppressive myeloid network that reinforces checkpoint signaling. More broadly in RCC, tumors may contain abundant intratumoral CD8^+^ T cells, yet higher CD8^+^ and CD4^+^ infiltrates have paradoxically been linked to higher tumor grade and shorter survival (187). RCC tumors can also show loss of MHC class I, which aligns with reduced effective CD8^+^ activity and a broader immunosuppressive pattern that includes Treg induction and reduced NK and dendritic cell function (188). Overall, obesity may make antitumor immunity less effective by changing the metabolic environment within RCC tumors. Excess lipids, altered glucose use, lactate buildup, and chronic inflammation can all place pressure on T cells – as these favor regulatory or suppressive immune responses. As a result, the presence of CD8^+^ T cells in the tumor does not always indicate an effective immune response. In some obesity-associated RCC tumors, these T cells may become functionally weakened rather than actively cytotoxic, which may partly explain poor responses to immunotherapy despite the presence of leukocytic infiltration.

### 11.2 CD4 T cells

CD4 T cells shape both anti-tumor and pro-tumor immunity by directing helper programs (such as Th1, Th2, and Th17) that support protective anti-tumor immunity and by generating regulatory T cells (Tregs) that suppress effector responses (189). In RCC, CD4^+^ T cells form a substantial fraction of the tumor immune infiltrate and appear in multiple phenotypic states within ccRCC tumors (182). Immune gene signature analyses across RCC subtypes show that different helper programs associate with outcome in distinct ways: one study found that a Th2 gene signature was the only immune signature associated with poor survival across clear cell, papillary, and chromophobe RCC (4), whereas another found that a Th17 gene signature correlated with prolonged survival in ccRCC only (189). These patterns suggest that T helper cell polarization influences prognosis, but in subtype- and context-specific ways.

Tregs, defined by expression of Foxp3, are a specialized CD4^+^ subset that limits immune activation (190). CD4^+^ T cells that constitutively express CD25 (CD4^+^CD25^+^Foxp3^+^) suppress the activity of other T cells as part of this regulatory program (190). In metastatic RCC patients, Tregs are increased in peripheral blood, and higher circulating Treg levels correlate with worse survival outcomes (190). Functionally, CD4^+^CD25^+^Foxp3^+^ Tregs isolated from the blood of mRCC patients inhibit effector T cell proliferation *in vitro*, confirming that they are not only numerically expanded but also actively suppressive (190). Tumor-derived signals can directly drive Treg generation. RCC tumors with high TGF-β production can convert CD4^+^CD25^-^ effector T cells into Tregs via TGF-β–linked mechanisms (191). In the Renca mouse model, blocking TGF-β in lung tissue reduces local Treg accumulation and lung metastases (192). Exosomes derived from RCC can express PD-L2 and preferentially accumulate in kidneys, lungs, and spleen (193). Interestingly, in immunodeficient mice lacking adaptive immunity, PD-L2+ exosomes suppress tumor growth and metastasis, whereas in immunocompetent hosts, PD-L2+ exosomes bind PD-1, increase Tregs, and reduce T effector proportions and activity in distant metastatic organs (193, 194).

These immune features help explain why obesity-associated RCC may combine persistent tumor-promoting inflammation with altered anti-tumor immunity, providing an important foundation for understanding the obesity paradox in RCC.

## 12 The obesity paradox in RCC

The obesity paradox describes situations where higher body mass is linked to better survival after a disease diagnosis, even though obesity increases the risk of developing that disease (195). Obesity is a well-established risk factor for developing RCC, yet many retrospective analyses of patient survival have reported better outcomes in patients with higher BMI at treatment initiation (22, 196, 197). This pattern initially suggested that RCC might follow a classic obesity paradox model, wherein excess body weight increases incidence but is protective for survival. The main clinical datasets that support this view and those that do not are summarized in **Tables 1**, **2**, respectively. Immunotherapy era datasets challenge a simple obesity paradox model. In several cohorts, obesity is associated with neutral outcomes, and in others it is associated with worse survival under ICI therapy (37, 40). RCC therefore does not demonstrate a single, consistent obesity paradox paradigm across treatment eras and regimens.

**Table 1:** Studies supporting an obesity paradox in RCC.

Study	RCC setting	Therapy type	Adiposity/metric	Main direction	Interpretation
Choi (196)	Non-metastatic RCC	Surgery	BMI categories (incl. ≥25)	Higher BMI associated with better survival	Higher BMI was associated with improved survival after RCC surgery.
Kaneko (198)	Localized ccRCC	Surgery	Visceral fat area (VFA)	Higher VFA predicts better RFS	Visceral obesity predicts *better recurrence-free survival* after curative surgery; BMI was not predictive in their analysis.
Naya (199)	Non-metastatic RCC	Surgery	Visceral fat area (VFA)	Higher visceral adiposity was associated with more favorable oncologic outcomes in this surgical RCC cohort.	CT-based visceral adiposity rather than BMI is associated with benefit.
Albiges (104)	Metastatic RCC	Targeted therapy (VEGF-TKIs)	BMI (≥25 kg/m^2^ grouping used in analyses/discussion)	High BMI associated with improved OS	Higher BMI is associated with longer survival, consistent with paradox under VEGF-targeted therapy.
Choueiri (200)	Metastatic RCC	Sunitinib or sorafenib	Obesity/BMI category	Obesity associated with longer OS	Obesity remains an independent favorable factor in multivariable models
Sanchez (34)	Metastatic ccRCC (COMPARZ) + primary RCC (TCGA)	TKIs (COMPARZ)	BMI ≥30 kg/m^2^	Obesity associated with longer OS after adjustment	Obesity shows a statistically supported survival benefit in COMPARZ after IMDC adjustment and also shows benefit in TCGA after clinicopathologic adjustment.
de Giorgi (197)	Metastatic RCC	Nivolumab after anti-angiogenic therapy	BMI + systemic immune-inflammation index	BMI <25 had worse OS than BMI ≥25, and remained independently adverse	BMI ≥25 is associated with better OS, while BMI <25 independently predicts worse OS. Worst outcomes occur with BMI <25 + high SII

RCC, renal cell carcinoma; ccRCC, clear cell renal cell carcinoma; BMI, body mass index (kg/m²); VFA, visceral fat area; CT, computed tomography; RFS, recurrence-free survival; OS, overall survival; VEGF, vascular endothelial growth factor; TKI, tyrosine kinase inhibitor; VEGF-TKIs, VEGF-targeted tyrosine kinase inhibitors; IMDC, International Metastatic RCC Database Consortium risk model; COMPARZ, the COMPARZ phase III trial; TCGA, The Cancer Genome Atlas; SII, systemic immune-inflammation index; NR, not reported.

**Table 2:** Studies not supporting an obesity paradox in RCC.

Study	RCC setting	Therapy type	Adiposity/metric	Main direction	Interpretation
Ladoire (201)	Metastatic RCC	First-line antiangiogenic agents (VEGF-targeted; incl. bevacizumab/sunitinib/sorafenib)	Visceral fat area (VFA) and subcutaneous fat area (SFA) (CT-based)	Higher VFA/SFA → worse outcomes (shorter TTP/OS)	Contradicts a paradox: greater adiposity predicted poorer survival in this anti-VEGF-treated mRCC cohort.
Bergerot (202)	Metastatic RCC	Targeted therapy + Immunotherapy (subgroup analysis)	BMI ≥25 kg/m^2^ vs <25 kg/m^2^	No obesity paradox under IO (trend favors lower BMI; NS)	While high BMI aligned with longer OS under VEGF-TKI/mTOR in this dataset, the immunotherapy subgroup does not support a paradox (inverse direction; not significant).
Boi (40)	Human RCC cohort plus orthotopic murine renal tumor model	Anti-PD-1-based immunotherapy	Obesity (BMI-defined; e.g., ≥30 kg/m^2^ vs <30 kg/m^2^); diet-induced obesity in mice.	In patients, obesity was associated with shorter PFS and OS after anti-PD-1 therapy; in mice, obesity reduced anti-PD-1-based therapeutic benefit	Does not support an RCC obesity paradox, but the mechanistic evidence for impaired immunotherapy response comes mainly from the preclinical model.
Sanchez (34)	Metastatic RCC	Immunotherapy cohort	BMI	Not significant after IMDC adjustment	Any BMI–OS association does not remain significant once IMDC risk is accounted for (i.e., not an independent “paradox” effect).
McManus (203)	Metastatic RCC	First-line dual immunotherapy (ipilimumab + nivolumab)	BMI; skeletal muscle index (SMI); subcutaneous adipose tissue index (SATI); visceral adipose tissue index (VATI) from baseline CT	Obese BMI (≥30 kg/m²) → worse PFS; high SMI → worse PFS; high SATI → worse PFS; no significant OS or ORR association overall	Does not support an obesity paradox in this first-line IO cohort: obesity was significantly associated with shorter progression-free survival, while no significant overall survival or objective response benefit was seen.

BMI, body mass index (kg/m²); VFA, visceral fat area; SFA, subcutaneous fat area; CT, computed tomography; VEGF, vascular endothelial growth factor; mRCC, metastatic renal cell carcinoma; PD-1, programmed cell death protein 1; PFS, progression-free survival; OS, overall survival; TTP, time to progression; IMDC, International Metastatic RCC Database Consortium risk model; IO, immunotherapy; TKI, tyrosine kinase inhibitor; NS, not significant; ccRCC, clear cell renal cell carcinoma.

A central issue is how adiposity is defined. Many studies use BMI ≥25 kg/m², which merges overweight and obesity into a single “high BMI” group. Other studies use BMI ≥30 kg/m², which isolates obesity itself. These different cutoffs change whether a survival advantage appears and how strong it looks (40, 204, 205). BMI also does not distinguish fat from muscle or capture fat distribution, so it can misclassify metabolic risk and confound associations with treatment efficacy (206). Individuals with BMI between 25 and 35 kg/m² may have high muscle mass, while others with marked visceral adiposity can fall within normal or only slightly elevated BMI ranges (206). Obesity itself spans metabolically healthy and metabolically unhealthy states, and inflammatory markers such as IL 6 and CRP vary widely within the same BMI category (207). These limitations support the view that many apparent obesity paradox outcomes may reflect a BMI measurement more than a uniform biologic benefit of obesity. **Tables 1**, **2** therefore serve as a framework to compare how BMI definitions, treatment context, and covariate adjustment shape the direction of reported associations.

### 12.1 Studies supporting an obesity paradox in RCC

Several RCC studies report outcome patterns that are compatible with an obesity paradox, especially in non-metastatic disease and in TKI treated metastatic RCC. In non-metastatic RCC, cohorts using BMI ≥25 kg/m² have found that higher BMI is associated with lower RCC specific mortality and better overall and recurrence free survival compared with BMI <25 kg/m² (196). A meta-analysis of non-metastatic RCC also reported improved outcomes in patients classified as obese versus non-obese (208). Imaging-based studies add nuance, with higher visceral fat area reported as an independent predictor of better survival and better recurrence free survival in localized disease (25, 198, 199). In **Table 1**, these studies appear within the surgery and non-metastatic group. In metastatic RCC treated with TKIs, higher BMI has repeatedly tracked with better survival. One analysis reported that BMI >25 kg/m^2^ was associated with longer overall survival in two large TKI-treated cohorts (104). In analyses from the COMPARZ trial and TCGA data, obesity defined as BMI ≥30 remained associated with longer overall survival even after adjustment for IMDC criteria (34). In a separate TKI treated cohort, higher BMI again associated with longer survival within the TKI subgroup (197). These TKI-era findings are captured in the metastatic RCC and targeted therapy rows of **Table 1** and illustrate a recurring pattern in which increased adiposity, especially when defined as BMI ≥25, coincides with better outcomes.

Immunotherapy-era data also include some study results that appear paradoxical. In one ICI treated metastatic RCC cohort (anti-PD-1 or anti-PD-L1 with or without anti-CTLA-4), obesity defined as BMI ≥30 kg/m^2^ was associated with longer overall survival in unadjusted analysis, although this advantage was lost after adjustment for IMDC risk (34). Another study reported that BMI >25 kg/m^2^ was associated with improved overall survival during ICI treatment, and a drop in BMI from >25 kg/m^2^ to <25 kg/m^2^ during therapy was associated with shorter survival (197, 209). Analysis of additional cohorts revealed that higher BMI or overweight status associated with better outcomes among patients who achieved clinical benefit, and that BMI <25 kg/m^2^, especially with high systemic immune inflammation, identified patients with the poorest prognosis under nivolumab after anti-angiogenic therapy (197). These ICI era results appear in the immunotherapy sections of **Table 1** and show that higher BMI can associate with better outcomes in selected RCC settings, particularly when overweight and obesity are combined and systemic inflammation is low.

### 12.2 Studies not supporting an obesity paradox in RCC

Many RCC studies do not support an obesity paradox, and some suggest the opposite pattern. In non-metastatic RCC, at least one imaging-based study reported that subcutaneous and visceral fat measures were not associated with overall survival (210), whereas other studies have reported that higher visceral fat area was associated with improved overall or recurrence-free survival (34). In metastatic RCC treated with TKIs, analysis of some cohorts showed no significant association between BMI and progression-free or overall survival, even when visceral fat area showed a prognostic signal (211). Another first line anti-angiogenic cohort reported that high visceral and subcutaneous fat area predicted shorter progression-free and overall survival, suggesting that larger fat depots can sometimes be associated with harm rather than benefit (201). These TKI and antiangiogenic null or adverse findings are listed in the upper rows of **Table 2**. Immunotherapy cohorts are even more heterogeneous and are captured in the ICI portion of **Table 2**. In the MSK immunotherapy cohort, an apparent obesity-associated survival advantage was not significant after adjustment for IMDC risk score (34). Two separate studies of ICI-treated metastatic RCC patients reported that an obesity paradox was not observed and that obesity was associated instead with worse progression free and overall survival (40, 203). A meta-analysis of ICI treated cancers concluded that RCC studies show contradictory results, with no consistent high BMI benefit (35). Metastatic RCC ICI studies have also not routinely incorporated CT-based measures of visceral or subcutaneous fat as predictors, so the role of fat distribution in ICI outcomes remains unclear (212). Of note, we compared outcomes in the same cohort with and without the inclusion of overweight individuals: obesity (BMI ≥30 kg/m²) was significantly associated with worse progression-free and overall survival, and this association persisted after adjustment for IMDC risk, age, and sex (40). When the BMI cutoff was shifted from ≥30 kg/m^2^ to ≥25 kg/m^2^, the survival curves for higher versus lower BMI nearly overlapped, indicating that combining overweight and obesity can mask obesity-specific adverse effects. Another ICI treated RCC cohort illustrated a trend toward worse survival at higher BMI that did not reach statistical significance, while the same researchers observed improved outcomes with higher BMI in TKI treated patients at the same institution (34). Together, the studies summarized in **Table 2** show that obesity can align with neutral, beneficial, or harmful survival patterns in RCC, depending on adiposity definition, treatment type, and analytical approach.

### 12.3 Biological mechanisms linked to the obesity paradox in RCC

The mixed results summarized in **Tables 1**, **2** have prompted several mechanistic explanations. One clinical explanation is that higher BMI can preserve fat and muscle stores, maintain nutritional status, and delay cancer associated cachexia, which can prolong survival independently of tumor cell biology (213). Another explanation is that RCC in patients with obesity may present with more favorable features, such as lower stage and lower grade (214). In one analysis, the survival advantage linked to higher BMI became non-significant after controlling for stage and grade, whereas in another the association with obesity remained even after adjustment for stage, grade, tumor size, and symptoms (215). These differences suggest that clinical factors explain part, but not all, of the BMI–outcome relationship observed in **Table 1**.

Metabolic mechanisms of the obesity paradox have focused on altered fatty acid pathways. Fatty acid synthase (FASN) has been highlighted, with lower FASN expression in tumors from patients with obesity associated with improved survival and higher FASN levels associated with worse survival (104). In support of these findings, pharmacologic inhibition of FASN reduces renal tumor cell growth in preclinical models (216). These findings support the idea that obesity-associated changes in lipid metabolism may “soften” tumor behavior in specific metabolic contexts. Although based on human melanoma tumor specimens, Hahn et al. found that obesity was associated with greater metabolic quiescence in tumor cells, characterized by decreased oxidative phosphorylation, a counterintuitive finding that stresses the need for continued study of this area (217). Transcriptomic analyses show that RCC tumors from obese and normal weight patients differ in expression of genes linked to hypoxia, angiogenesis, and epithelial mesenchymal transition, even though overall immune infiltration and tumor mutational burden are not consistently different (34). In the COMPARZ trial cohort, PD-L1 expression was lower in RCC tumors from patients with obesity than in those from normal weight patients (34), suggesting lower levels of PD-L1/PD-1–dependent suppression of CD8^+^ T cell tumor immunity. Peritumoral adipose tissue in obesity shows increased leukocyte infiltration and greater hypoxia, and has been proposed as a local leukocyte reservoir during checkpoint therapy (57), which could impact ICI outcomes. These observations provide a biological backdrop for the TKI and ICI patterns reported in **Tables 1**, **2**.

Obesity-driven alterations in immune mechanisms may also help explain why obesity does not translate into a uniform survival advantage, although it is important to note that several of the supporting preclinical studies were performed outside ccRCC models. In one study, obesity increased frequencies of PD-1^+^ CD8^+^ T cells in B16 melanoma tumors, reflecting patterns seen in the peripheral blood of healthy humans (218); these data suggest that obesity increase tumor dependence on PD-1 signaling making them more sensitive to blockade. However, Khojandi et al. found no increased PD-1 expression on CD8^+^ TILs from a mixed cohort of melanoma and breast cancer patients as BMI increased (219). In patients with RCC, we found that obesity was associated with reduced frequencies of activated PD-1^high CD8^+^ TILs (40). Together, these results suggest that the immune effects of obesity on CD8^+^ TIL function may be tumor-context dependent.

One notable clue to the variable results obtained to date was provided by De Giorgi et al., who reported that in a ccRCC nivolumab cohort, low systemic inflammation was associated with better survival across BMI strata, with the worst survival occurred in patients with both high inflammation and low BMI (197). This supports the idea that an inflammatory state can dominate over other BMI signals and may underlie some of the discordant results across the studies in **Tables 1**, **2**. Overall, current literature suggests that obesity in RCC should not be treated as a single biologic entity or a reliable predictor of improved outcome. BMI is a crude surrogate that mixes fat and muscle and ignores fat distribution and the individual’s metabolic health. Obesity related biology likely influences tumor metabolism, angiogenesis, peritumoral inflammation, and immune cell function, but these effects are context dependent and treatment dependent. The contrasting findings in **Tables 1**, **2** argue for more precise measures of body composition, inflammatory status, and immune phenotype to clarify when adiposity confers risk, when it coincides with benefit, and how it affects immunotherapy outcomes in RCC.

## 13 Therapeutic implications for obesity-associated RCC

Immune checkpoint inhibitors are now central to the clinical management of RCC. Combinations that pair PD-1/PD-L1 blockade with anti-angiogenic agents or other immunotherapies are used as first line options for many patients (220). Clinical benefit from nivolumab-based regimens is seen across PD-L1 expression levels, so PD-L1 status alone does not reliably define who should or should not receive these therapies (221). At the same time, obesity cannot be viewed as a uniform modifier of ICI efficacy in RCC (**Tables 1**, **2**). The conflicting patterns observed to date suggest that collapsing BMI into a single “high versus low” variable can mix distinct biological groups and distort interpretation. Taken together, these RCC-specific inconsistencies support moving toward a therapeutic approach that treats any “obesity-associated benefit” as context dependent, rather than assuming a default survival advantage when selecting or sequencing ICIs. Therefore, therapy development using a biomarker-based strategy is essential.

However, no single biomarker consistently predicts ICI benefit in RCC across trials and platforms (220). PD-L1 immunohistochemistry is limited by intratumoral and inter site heterogeneity, dynamic changes with treatment, and lack of assay standardization, which restrict its value as a standalone decision tool (222). Exploratory molecular classifiers offer a more mechanistic framework. A high T effector gene signature is associated with better outcomes on atezolizumab (anti-PD-L1) plus bevacizumab (anti-VEGF), whereas a high myeloid inflammation gene expression signature is associated with lower progression free survival in patients on atezolizumab-containing study arms, but not those on a sunitinib (TKI) study arm (223). Obesity-related immune rewiring can plausibly intersect with these transcriptomic patterns. In tumor-free adults, obesity is frequently associated with elevated systemic inflammatory mediators such as IL-6, IL-17, C-reactive protein (CRP), and prostaglandin E_2_, and under stimulation this inflamed state can amplify pro-angiogenic outputs such as VEGF (224). De Giorgi et al. showed that high systemic inflammation independently predicted worse overall survival in nivolumab-treated ccRCC: patients with both high inflammation and BMI <25 kg/m² had the worst survival (197). These findings support the idea that inflammation can outweigh any apparent BMI-related survival advantage in RCC (197). Building on this concept, patients with high systemic inflammation and strong myeloid-inflammatory signatures in the tumor may not be ideal candidates for ICI monotherapy. These patients may benefit more from combination approaches that first reduce myeloid-driven suppression, block inflammatory cytokine signaling, or target angiogenesis. By contrast, patients who retain a stronger T-effector profile and show lower levels of myeloid inflammation may be more likely to respond to standard ICI-based combinations. In this way, immunometabolic profiling could guide whether checkpoint blockade is used alone, combined with anti-angiogenic therapy, or paired with myeloid- or cytokine-targeted treatments.

In renal cancer cohorts, obesity has been linked in some settings to stronger intratumoral angiogenesis signals and altered checkpoint phenotypes, but the direction and magnitude of these effects vary between studies (34). This variability highlights that obesity-associated biology in RCC is heterogeneous and should be integrated into biomarker frameworks rather than assumed to act in a single direction. This heterogeneity strengthens the rationale for myeloid-targeting strategies as partners for checkpoint therapy when obesity-associated myeloid programs are prominent (195). In the Renca renal cancer model, obese non-responders to therapy show a weak CD8^+^ T cell signature together with a strong myeloid signature and elevated intratumoral IL-1β (40). These findings support combination strategies that jointly target myeloid recruitment or polarization alongside ICIs. Such approaches could limit macrophage infiltration or reprogram macrophages away from immunosuppressive, tumor permissive states that are associated with poor prognosis in RCC (225). Targeting inflammatory lipid pathways is also a relevant approach, because prostaglandin E_2_ is elevated as part of obesity-linked systemic inflammation in humans (224). This supports placing COX-2 and PGE_2_ directed approaches within a broader myeloid-targeting toolbox, rather than treating them as isolated interventions. Cytokine directed strategies may also be best viewed within a network rather than as single axis interventions (195). Obesity-associated increases in IL-6 and IL-17 occur alongside lipid mediator shifts such as PGE_2_, and these combined changes interact with angiogenesis and myeloid activation rather than mapping cleanly onto one cytokine (224). RCC microenvironments can also maintain immunosuppression through cytokines such as IL-1β, TGF-β and IL-10 (226). This supports multi-pronged combinations that consider cytokine blockade, checkpoint inhibition, and myeloid remodeling in parallel. For example, future trials could prospectively evaluate whether RCC patients with high BMI plus inflammatory-myeloid signatures benefit more from ICI combined with IL-1β/IL-6 pathway blockade, COX-2/PGE_2_ targeting, or MDSC-directed therapy than from ICI-based therapy alone.

## 14 Knowledge gaps and conclusions

Several major knowledge gaps limit how effectively obesity can be integrated into therapy development and clinical care for patients with RCC. The first gap is model selection. Obesity effects on anti-tumor immunity and immunotherapy are not uniform across tumor types, and the same obese host state can produce opposing therapeutic outcomes depending on tumor biology, immune dependencies, and microenvironment characteristics. RCC-relevant obese pre-clinical models are therefore essential. In the Renca model, the combined presence of diet-induced obesity and renal cancer demonstrate that overall immunotherapeutic efficacy is reduced in obese hosts, even when baseline intrarenal tumor burden is similar at treatment initiation (40). In this model, obese therapy non-responders retained immune signatures that resembled treatment-naive tumors, whereas individual lean and obese therapy responders showed beneficial immune remodeling after therapy (40). This suggests that obesity can block the shift into a “treated” immune state rather than simply altering baseline immune composition within tumors. Including obesity-resistant controls represented by mice that receive the same obesogenic diet but do not develop obesity is a practical design feature (227). These controls separate diet composition effects from the obese host phenotype and should be more routinely built into RCC obesity model pipelines.

A second gap is identifying obesity-related biomarkers beyond BMI. BMI is an imprecise surrogate for studying the biology of adiposity, and RCC cohorts show that survival associations change when overweight and obesity are analyzed separately or when models adjust for IMDC risk group and sex. Human renal tumor datasets also show that obesity-associated differences in leukocyte composition can be subtle or cohort dependent, so BMI categorization alone cannot reliably infer the immune state that will shape treatment response (34). These findings support moving toward obesity-related biomarkers that are based upon body composition and systemic inflammation more directly. In tumor-free adults, obesity is frequently linked to elevated CRP and lipid mediators such as PGE_2_, which may influence tumor immunity and angiogenesis once RCC develops. In addition, existing RCC-specific biomarkers also need refinement, especially in the context of host obesity. PD-L1 testing is limited by spatial heterogeneity, sensitivity to prior therapies, and assay variability, which weakens reproducibility and cross-study comparisons (222). It is not yet clear whether or how obesity alters PD-L1 expression in RCC. Molecular and genomic markers provide more mechanistic therapeutic candidates. T cell effector and myeloid inflammation gene-expression signatures varied by treatment arm in IMmotion151, a phase 3 trial in previously untreated metastatic RCC patients, and genomic features such as PBRM1 loss and frameshift indel burden have been linked to nivolumab benefit in specific cohorts (223, 228, 229). These approaches could be adapted to map obesity-associated immune states more precisely than is possible with BMI alone.

A third gap lies in clinical trial design. Even in the broader RCC ICI literature, trial interpretation is complicated by heterogeneous populations and variable PD-L1 analyses, which makes cross study synthesis difficult (230). Obesity adds another layer of complexity, because renal cohorts show that results change depending on how obesity is defined, whether BMI is dichotomized at 25 or 30 kg/m², and whether overweight and obesity are pooled or separated. Future RCC trials that aim to understand obesity-associated immune states should report body composition measures such as visceral fat, sarcopenia, or waist circumference, along with inflammatory markers, in addition to BMI. They should also pre-specify stratification schemes based on immunometabolic phenotype rather than assuming BMI captures the relevant metabolic state. T-effector and myeloid inflammation gene-expression signatures, which are already linked to different outcomes between treatment types, could be used to stratify RCC patients by immune phenotype in immunotherapy trials. A potential translational strategy would be to categorize RCC patients for combinatorial therapies that target immune and metabolic deficiencies rather than relying on BMI alone. Patients could first be stratified by CT-defined obesity patterns, including visceral-predominant versus subcutaneous-predominant adiposity. This could then be combined with biopsy-based and serum-based profiling to define treatment-relevant phenotypes: T-effector-high, myeloid-inflamed, angiogenic-hypoxic, and lipid-enriched inflammatory RCC. T-effector-high tumors should not be defined by CD8^+^ T cell infiltration alone, but by a functional cytotoxic/IFN-γ gene program, including markers such as granzyme B (GZMB), perforin 1 (PRF1), interferon-gamma (IFNG), C-X-C motif chemokine ligand 9 (CXCL9), T-box transcription factor 21/T-bet (TBX21), and eomesodermin (EOMES). These tumors may be more suitable for ICI-based combinations when myeloid suppression and hypoxia are not dominant. Myeloid-inflamed tumors would show increased MDSCs, TAMs, CD163^+^ macrophages, IL-1β, and IL-6. In this subgroup, ICI plus IL-1 pathway blockade could be tested in biomarker-selected trials, with anakinra included as a clinically available IL-1 receptor antagonist rather than an established RCC therapy (135, 231). Angiogenic-hypoxic tumors would show high VEGF/VEGFA expression, VEGFR pathway activity, HIF-related markers, carbonic anhydrase IX (CAIX), GLUT1, or other hypoxia-associated vascular programs. These features would support treatment strategies that combine ICI with VEGF/VEGFR TKIs, anti-VEGF therapy, or HIF-2α inhibition. Lipid-enriched inflammatory tumors would be characterized by visceral adiposity, abnormal lipid or adipokine profiles, increased CD36, FABP5, PLIN2, SCD1, FASN, or ACLY, and enrichment of lipid-associated or CD163^+^ macrophages. These tumors may be candidates for trial designs testing ICI with clinically available lipid-modifying approaches, for example statin-based lipid lowering in patients with dyslipidemia. This type of multi-parameter biomarker use could identify patients most likely to respond to ICI-based therapy alone versus those who may need ICI combined with anti-angiogenic, cytokine-directed, myeloid-directed, or lipid-metabolic therapy. Turning the “obesity paradox” in RCC into actionable treatment strategies will require better models, multi-parameter biomarkers that better inform clinical practice, and trials that more accurately define adiposity and the overall metabolic state in patients.
